# Molecular characterization of *Streptococcus agalactiae* isolated from pregnant women and newborns at the University of Gondar Comprehensive Specialized Hospital, Northwest Ethiopia

**DOI:** 10.1186/s12879-020-4776-7

**Published:** 2020-01-13

**Authors:** Mucheye Gizachew, Moges Tiruneh, Feleke Moges, Mulat Adefris, Zemene Tigabu, Belay Tessema

**Affiliations:** 1 Department of Medical Microbiology, School of Biomedical and Laboratory Sciences, College of Medicine and Health Sciences, University of Gondar, P. O. Box 196, Gondar, Ethiopia; 2 Department of Gynecology and Obstetrics, School of Medicine, College of Medicine and Health Sciences, University of Gondar, P. O. Box 196, Gondar, Ethiopia; 3 Department of Pediatrics, School of Medicine, College of Medicine and Health Sciences, University of Gondar, P. O. Box 196, Gondar, Ethiopia

**Keywords:** GBS, Molecular characterization, Newborns, Pregnant women, Serotype, Sequence type

## Abstract

**Background:**

*Streptococcus agalctiae* (Group B Streptococcus, GBS) is a perinatal pathogen and a leading cause of neonatal infections worldwide. Serotype, sequence type, clonality, antibiotic resistance genes and surface protein profiles of GBS are scarce in Ethiopia, a reason that this study was planned to investigate. .

**Methods:**

Sixteen colonizing GBS isolates obtained from recto-vaginal swabs of pregnant women and body surfaces of newborns were further analyzed. Minimum inhibitory concentration (MIC) test, and whole genome sequence (WGS) methods were done for antibiotic susceptibility test, and molecular characterization of the isolates.

**Results:**

All the GBS isolates analyzed were belonged to four capsular serotypes: II, 11/16(68.8%), V, 3/16(18.8%), Ia and VI each with 1/16(6.3%) and five sequence type (ST-2, ST-10, ST-14, ST-569 and ST-933). Sequence type-10 was the most predominant ST followed by ST-569. The five STs were grouped into the four clonal complexes (CC - 1, CC-10, CC-19, and CC-23). Different surface proteins and pili families such as ALP1, ALPHA, ALP23, PI-1 / PI-2A1, PI-1 / PI-2B, and Srr1 were detected from WGS data. All isolates were found to be susceptible to the tested antibiotics except for tetracycline in MIC and WGS test methods used. Tetracycline resistant determinant genes such as TETM and TETL / TETM combination were identified.

**Conclusion:**

Further studies on serotype and molecular epidemiology will provide a comprehensive data of the GBS capsular serotype and clones available in Ethiopia.

## Background

Group B Streptococcus (GBS) or *Streptococcus agalactiae* remains a leading cause of neonatal sepsis, pneumonia and meningitis, often associated with high morbidity and mortality in Europe, USA, and Australia even though there was a wide use of intrapartum antibiotic prophylaxis (IAP) since the mid 1990s. However, molecular profiles of GBS in many countries outside of these regions was not well documented [1, 2]. GBS causes early-onset neonatal disease (EOD) which is characterized by onset of clinical symptoms during the first week of life (0–6 days) following birth; it usually results in vertical transmission from colonized pregnant women to newborns during or just before delivery [3]. The other form of GBS disease appears in infants is the late-onset disease (LOD) - characterized by the onset of symptoms within 7 days to 89 days of age. It can be acquired from the mother or environmental sources [4, 5]. It is also an important cause of preterm delivery, stillbirth, and puerperal sepsis [6, 7].

To colonize and cause diseases, GBS use a collection of virulence factors; one of the most important being a capsular polysaccharide (CPS) [8]. Human GBS has currently 10 serotypes, Ia, Ib, II to IX, based on serotype specific antigens [9] and the current candidate capsular polysaccharide conjugate vaccines target only a subset of these [10]. A study described that the five most common serotypes, Ia, Ib, II, III, and V accounted for more than 85% of serotypes in global regions that have reported serotype data, including the Americas (96%), Europe (93%), and the Western Pacific (89%) [11], and Africa (91.8%) [12]. A review demonstrated that among the ten serotypes, Ia, Ib, II, III, and V accounts for 98% of the identified colonizing isolates worldwide. Serotype III, associated with invasive disease, accounts for 25%, but is less frequent in some South American and Asian countries, but serotypes VI–IX are more common in Asia [13]. Another report from Iran showed that serotypes V (19.6%), II (12.5%) and IV (12.5%) were the most frequent followed by serotypes III (10.7%) and VI (10.7%), Ib (8.9%), Ia (7/1%), VII (5/3%) and VIII (5/3%); and 7.1% of strains were non-type-able [14]. A study in Ghana showed as serotypes VII (38.5 to 42.9%) and IX(26.9 to 32.1%) were the most common serotypes [15]. Serotyping of GBS is useful to understand the local epidemiology, for monitoring of serotype replacement or capsular switching, and for contributing existing serotype profiles in the area for rational, effective and broad serotype coverage GBS vaccine development [16, 17]. But, there is scarcity of serotype profile data of GBS colonizing pregnant women and newborns in Northwest Ethiopia for the last three decades.

GBS remained susceptible to the beta-lactam antibiotics, but, resistance to macrolides, lincosamides, fluoroquinolones and other antibiotics used as alternative therapy has been reported [18]. Macrolide resistance in GBS is represented by two mechanisms: Target Site Modification by Erythromycin ribosomal methylase, mediated by ermB, ermA, ermTR, or ermC genes which confers cross resistance to macrolides, lincosamides, and streptogramin_B_ (MLS_B_ phenotype) [19]. Erm genes encode methylase 23S rRNA, which is responsible for methylation of erythromycin and clindamycin receptor sites in ribosomes [20]. This resistance can be constitutive macrolide lincosamidestreptogramin_B_ (cMLS_B_) resistance, as well as inducible macrolide lincosamidestreptogramin_B_ (iMLS_B_) resistance [21]. The second resistance mechanism that found in GBS is Macrolide efflux pump, mediated by mef genes, which confers resistance to 14 and 15 member macrolides only. In addition, a novel efflux system distinct from the Mef pump and encoded by mreA (for macrolide resistance efflux) was reported in a unique strain of *S. agalactiae* COH31 γ/δ [22]. Susceptible GBS isolates also showed to possess the mreA gene, and it might function as a housekeeping gene [23].

Regarding the study of antimicrobial resistance markers (genes), a study revealed that tetM was the most frequently (84%) identified in all groups. Macrolide resistance genes were found in a small proportion of the isolates (8%) and each of the three relevant genes was represented as 3.3% ermB, 2.5% ermA, and 2.3% mefA/E. In addition, one isolate contained both ermA and ermB, and one contained both ermA and mefA/E [24]. In another study, macrolide resistance genes were screened and the resistant rate in total isolates was reported as 69.5%. Ribosome methylation genes (*erm*B, *erm*A/TR, *erm*C) were screened in all examined isolates and the most prevalence gene (63.04%) was *erm*B,and *erm*A\TR gene accounted 23.9%, but *erm*C gene was not detected. Findings of the study displayed that the prevalence rate of efflux pumps encoding genes (*mef*A and *mreA*) were 8.69 and 69.5% respectively. The study also showed that 14 (30.4%) of the isolates were not recognized as a carrier of any erythromycin resistance genes. One isolate among the screened isolates was harbored four erythromycin resistance genes except *erm C* gene [25]. Muller et al. reported that resistance to erythromycin and clindamycin was found in 8 and 7%, respectively. Macrolide resistance genes mef(A), erm (TR) and erm(B) were found in one, two, and five isolates, respectively; only 5/8 (62.5%) of the isolates exhibited both genotypic as well as phenotypic resistance. One genotype occurred in 36% of the subset [26].

A study detailed that GBS has different surface proteins such as alpha, Alp1, Alp2, Alp3, Alp4, and Rib. These proteins are collectively named as alpha-like protein (Alp) family [21, 27]. The biological role of Alp proteins is not well documented though they are targets for protective antibodies demonstrated in the animal model. GBS uses alpha protein to invade the epithelial cells by interacting with host cell glycosaminoglycans [21, 28, 29]. Genetic profiles of GBS strains obtained from around the globe is investigated by different molecular typing methods including multi-locus sequence type (MLST) [21, 27, 30]. However, there is scarcity of data regarding to the antimicrobial resistance gene markers plus sequence types and surface protein profiles of GBS in Northwest Ethiopia. Therefore, this study was aimed to determine the serotypes, antibiotic resistance genes, sequence types and surface protein profiles of GBS recovered from pregnant women and newborns in Northwest Ethiopia.

## Methods

### Study setting

It was conducted at the University of Gondar Comprehensive Specialized Hospital. Northwest Ethiopia. The hospital serves more than 5, 000,000 people and it has about 450 to 600 pregnant women admission services a month. So far, there is no routine screening of pregnant women and provision of IAP for GBS has been established in the hospital [31].

### *Streptococcus agalaciatae* isolates and serotype determination

Colonizing GBS isolates were recovered from recto-vaginal area of pregnant women at time of delivery and body surfaces of newborns immediately following delivery and stored in deep freezer (− 80 °C). The GBS isolates were randomly picked from a stored collection in a deep freezer. Of these, ten were from recto-vaginal swabs of pregnant women, and six were from the newborns (three nasal, two eye and one umbilical swabs). The isolates were further processed by using streptococci Lancefield grouping GBS latex slide agglutination kits and short-read whole genome sequencing (WGS) as previously described [32]. This study protocol was in accordance with the ethical standards of the responsible regional committee on human experimentation and the Declaration of Helsinki of 1975 (revised in 1983). It was approved by the ethics committees at the University of Gondar. Informed written consent was obtained from the pregnant women and care providers of newborns enrolled in this study.

### Conventional minimum inhibitory concentration (MIC) determinations

Sixteen isolates were subjected to broth micro-dilution testing (BDT) for determination of Minimum Inhibitory Concentration **(**MIC) and compared it with WGS-based characterization of antibiotic susceptibility patterns including resistance genes in the GBS isolate. Antibiotics included six β-lactams (ceftizoxime, cefoxitin, cefotaxime, cefazolin, ampicillin and penicillin), erythromycin, clindamycin, tetracycline, levofloxacin, ciprofloxacin, daptomycin, vancomycin and linezolid) were tested. A well containing both erythromycin and clindamycin detected inducible clindamycin-resistance, as previously described [33]. In addition, these isolates were test for their antimicrobial susceptibility test by using the disc diffusion method as per the CLSI, 2014 guideline [34].

### Whole genome sequencing

‘The WGS of GBS was performed at the CDC streptococcus laboratory in the Atlanta, Georgia (USA), and drug resistant genes, serotypes, sequence types, clonal complexity and surface protein profiles were predicted from the WGS data. GBS colonies were cultured on Todd Hewitt Broth with 0.5% yeast and incubated overnight at 37 °C in 5%CO_2_. Genomic DNA for WGS was extracted manually using a modified QIAamp DNA mini kit protocol (Qiagen, Inc., Valencia, CA, USA). Nucleic acid concentration was quantified by an Invitrogen Qubit 3.0 Flurometer assay (Thermo Fisher Scientific Inc.,Waltham, MA, USA) and samples were sheared using a CovarisM220 ultrasonicator (Covaris, Inc.,Woburn, MA, USA) programmed to generate 500-bp fragments. Libraries were constructed on theSciCloneG3 (PerkinElmer Inc., Waltham, MA, USA) using a TruSeqDNA PCR-Free HT library preparation kit with 96 dual indices (Illumina Inc., San Diego, CA, USA) and quantified by a KAPA qPCR library quantification method (Kapa Biosystems Inc., Wilmington, MA, USA). WGS was generated employing MiSeqinstrument and the MiSeq v2 500 cycle kit (Illumina Inc).WGS-based identification of serotypes employed the query DNA sequences described in the Additional file 1.

To identify the clonality, the sequence types (STs) of GBS isolates were investigated using the seven house-keeping genes: adhp, atr, glck, glna, phes, sdha and tkt by comparing with the standard references available at the MLST 1.8 database (https://cge.cbs.dtu.dk//services/MLST/). To visualize the possible evolutionary relationships between isolates, STs of the study isolates and the globally reported strains were computed using PHYLOViZ software v2.0 based on Global optimal eBURST (goeBURST) algorithm [35].

## Results

As indicated in Table 1, the genomes size of the GBS ranged from 2,018,343 to 3,891,488 bp. The numbers of contig in the DNA sequences per genome ranged from 29 to 1797.Of the 16 isolates sequenced, four serotypes were identified as, serotype II, 11/16(68.8%), V, 3/16(18.8%), Ia and VI each with 1/16(6.3%). Capsular polysaccharide serotypes II and V were accounted for 14/16(87.5%). Capsular serotype II accounted for 5/6(83.3%), and 6/10(60.0%) among the neonatal and pregnant women isolates analyzed respectively. Antibiotic susceptibility testing was performed for all 16 GBS isolates by using MIC. Results have shown that, they were sensitive to all antibiotics tested except for tetracycline to which 93.8% of GBS isolates showed resistance Antibiotic susceptibility test results from sequenced data also showed that all isolates were susceptible except for tetracycline to which the 93.8% isolates showed resistance. Tetracycline resistance was predominantly due to tetM, which was detected alone in the 56.3% of the isolates, or in 37.5% of the isolates in association with tetL. However, there are no genes encoding drug resistance detected for other antibiotics tested. In addition, penicillin binding protein (PBP1A and PBP2X) for beta-lactam antibiotic was not detected in our isolates. Of the surface proteins screened from our isolates, the alpha-like protein (ALPH), serine-rich repeat-1 (SRR1),and Pili, were detected. Among the ALPH families, 43.8% of the isolates had ALPHA, followed by ALP1 (25.0%), and all isolates harbored at least one pilus islands (PI), of which, the PI1/PI2A1 combination was the most prevalent sub-family of Pili that was identified among the 14/16 (87.5%) isolates (Table 1).

**Table 1:** Molecular characterization of GBS (n-16) isolated at the University of Gondar Comprehensive Specialized Hospital, Northwest Ethiopia

Isolate ID	Serotypes	Sequence type	Clonal complex	Year of Isolation	Size (bp)	contigs	N50	Drug resistant genes	Surface protein genes			Allelic profiles						
									ALPH	SRR	Pili	adhP	pheS	atr	glnA	sdhA	glcK	tkt
1451–19	V	2	19	2017	NA	NA	NA	TETM	ALP1	SRR1	PI1:PI2A1	1	1	3	1	1	2	2
1451–20	V	2	19	2017	2,123,519	36	154,151	TETM	ALP1	SRR1	PI1:PI2A1	1	1	3	1	1	2	2
1451–21	V	2	19	2017	2,126,782	29	136,443	TETM	ALP1	SRR1	PI1:PI2A1	1	1	3	1	1	2	2
1451–22	II	K810	10	2017	2,049,638	60	85,692	TETL:TETM	ALPHA	SRR1	PI1:PI2A1	9	1	4	1	3	3	2
1451–23	II	10	10	2017	2,031,351	71	74,284	TETL:TETM	Neg	SRR1	PI1:PI2A1	9	1	4	1	3	3	2
1451–24	II	10	10	2017	2,065,816	100	65,164	TETL:TETM	Neg	SRR1	PI1:PI2A1	9	1	4	1	3	3	2
1451–25	II	10	10	2017	2,033,318	78	82,248	TETL:TETM	neg	SRR1	PI1:PI2A1	9	1	4	1	3	3	2
1451–26	II	10	10	2017	3,891,488	1797	6059	TETL:TETM	neg	SRR1	PI1:PI2A1	9	1	4	1	3	3	2
1451–27	II	10	10	2017	2,043,421	58	85,697	TETL:TETM	ALPHA	SRR1	PI1:PI2A1	9	1	4	1	3	3	2
1451–28	VI	14	1	2017	2,116,176	29	137,184	TETM	ALP1	SRR1	PI1:PI2B	1	1	2	1	5	2	2
1451–29	II	569	10	2017	2,022,186	40	150,703	TETM	ALPHA	SRR1	PI1:PI2A1	9	1	1	1	3	1	2
1451–30	II	569	10	2017	2,018,612	36	111,901	TETM	ALPHA	SRR1	PI1:PI2A1	9	1	1	1	3	1	2
1451–31	II	569	10	2017	2,086,254	279	108,514	TETM	ALPHA	SRR1	PI1:PI2A1	9	1	1	1	3	1	2
1451–32	II	569	10	2017	2,018,343	40	138,563	TETM	ALPHA	SRR1	PI1:PI2A1	9	1	1	1	3	1	2
1451–33	II	569	10	2017	2,023,055	36	150,759	TETM	ALPHA	SRR1	PI1:PI2A1	9	1	1	1	3	1	2
1451–34	Ia	933	23	2017	2,082,078	97	166,116	neg	ALP23	SRR1	PI1:PI2A3	5	4	6	1	2	1	81

**Fig. 1:**
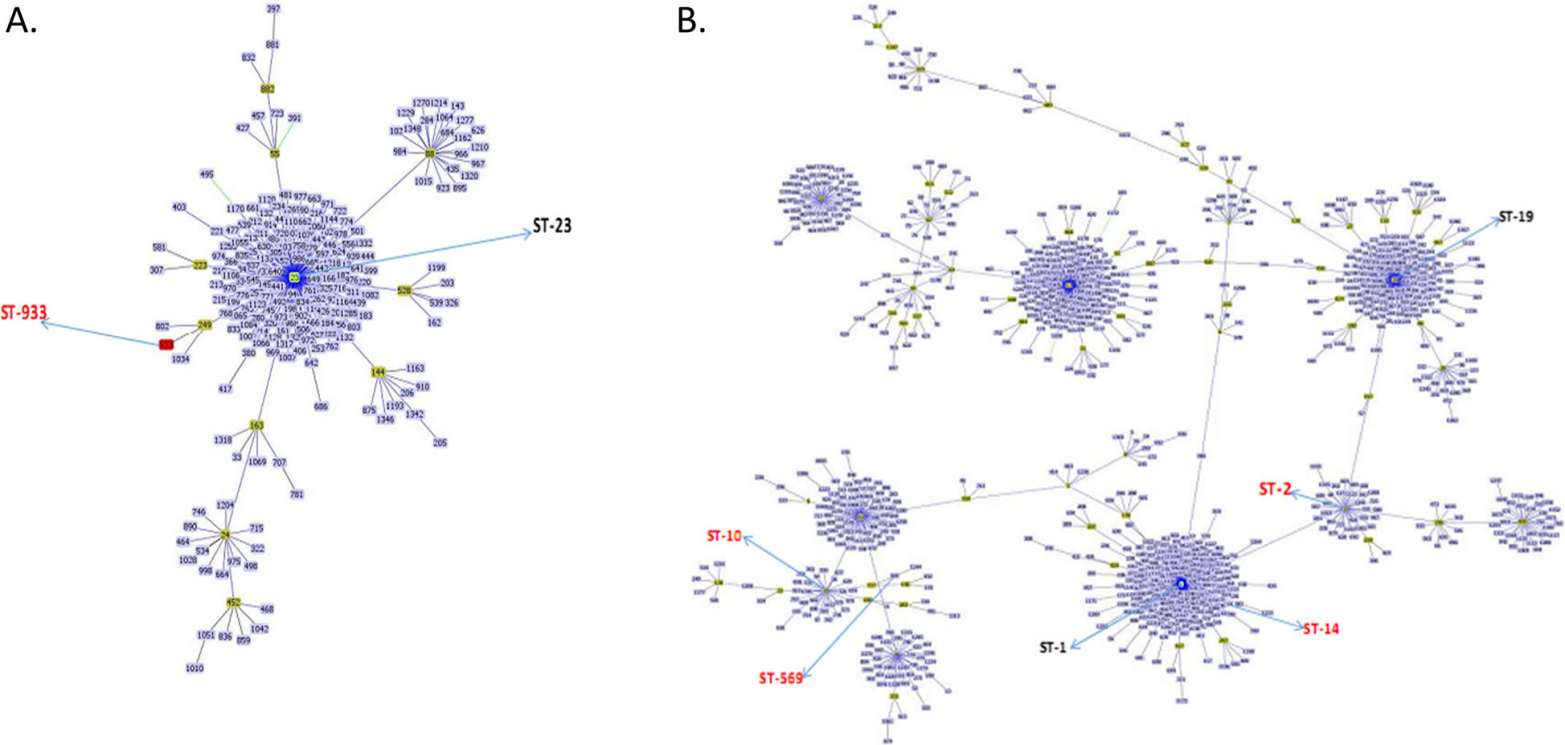
Global optimal eBURST (goeBURST) algorithm for Group B Streptococcus was calculated by using PHYLOViZ softwarev2.0. The figure depicts, (**A**) ST-933 (RED) was belonging to CC-23 (deep BLUE center) with ST-23 as founder ST, and (**B**) ST-10 and ST-569 were belonging to CC-10 with ST-10 as founder ST, ST-14 was belonging to CC-1, and ST-2 was belonging to CC-19.The primary founder of a group is the ST that differs from the largest number of other STs at only a single locus (i.e. the ST that has the greatest number of single-locus variants; SLVs). In the goeBURST diagram, the circle representing the predicted primary founder is colored blue and the areas of each of the circles indicate the prevalence of the ST in the input data. The STs that are subgroup founders (ST-2), according to the default definition of a subgroup, or a user-defined definition, are colored yellow on the goeBURST diagram

The MLST data reveals that the study isolates showed five sequence types (STs), and four clonal complexes (CCs) which are of different phylogeny and is suggestive of different clones circulating in Northwest Ethiopia. Overall, the most common STs were ST-10 (37.5%) followed by ST-569 (31.3%). Sequence type (ST)-14 was represented by a single GBS isolate. The predominant CCs were CC-10 (68.8%) followed by CC-19 (18.8%). Clonal complex 1 and 23 each was represented by a single isolate. goeBURST reveals about 68 clonal complexes from the available datasets globally. The goeBURST diagram shows that the sequence types STs-10 and STs-569, a double locus variant (DLV) of ST-10 (Fig. 1b), from the study isolates was belonging to clonal complex (CC) 10 with ST-10 as founder ST, whereas, ST-2, a subgroup founder, was belonging to the CC-19, ST-14 was grouped into the CC-1, and ST-933 was categorized into the CC-23 (Table 1, and Fig. 1a).

**Fig. 2:**
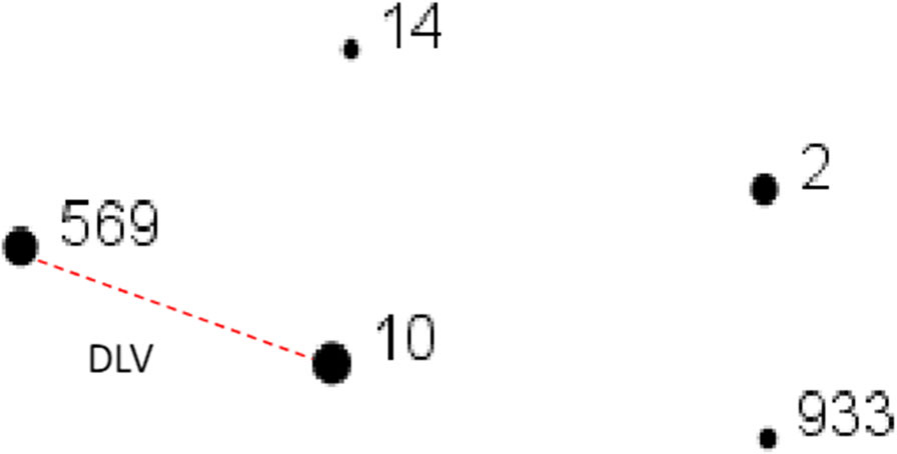
ST-569 and ST-10 showed double locus variant (DLV) indicated in RED broken line. The size of the dots or the diameter of the circle indicates the number of the STs included

In addition, Fig. 2 showed that ST-569 and ST-10 were in the double locus variant (DLV), where as the rest of the STs were with the single locus variants (SLV), the STs those which were directly connected to the founder and they differ from the founder by only one single locus or allele. The size of the black dots indicate the number of the isolates included in the specified STs in which the larger the size, the higher the number of the isolates included. ST-14, and ST-933 were 1/16(6.25%) each, but STs-10 were 6/16 (37.5%).

As it is shown in Table 2, the 16 GBS isolates were tested for about 15 different antibiotics by using either disc diffusion, minimum inhibitory concentration (MIC) or short-read whole genome sequencing methods. Certain isolates showed resistance to different antibiotics tested ranging between 1(6.25%) for penicillin to 14(87.5%) for tetracycline in the disc diffusion test method.

**Table 2:** Antibiotic susceptibility test results of GBS isolates (*n* = 16) at the University of Gondar Comprehensive Specialized hospital, Northwest Ethiopia

S/No.	Antibiotics tested	Interpretations	Test methods used		
			Disc diffusion, N(%)	MIC, N(%)	WGS, N(%)
1	Penicillin (PEN)	S	15 (93.75)	16 (100)	16 (100)
		R	1(6.25)	0	0
2	Ampicillin (AMP)	S	16 (100)	16 (100)	16 (100)
		R	0	0	0
3	Clindamycin (CLY)	S	14(87.5)	16 (100)	16 (100)
		I	1(6.25)	NA	NA
		R	1(6.25)	0	0
4	Erythromycin (ERY)	S	14 (87.5)	16 (100)	16 (100)
		R	2(12.5)	0	0
5	Vancomycin (VAN)	S	13 (81.25)	16 (100)	16 (100)
		R	3(18.75)	0	0
6	Ciprofloxacin (CIP)	S	10 (62.5)	16 (100)	16 (100)
		R	6(37.7)		0
7	Ceftriaxone (CFX)	S	12(75)		NA
		R	4(25)		
8	Levofloxacin (LEVO)	S	NA	16 (100)	16 (100)
		R	NA	0	0
9	Daptomycin (DAP)	S	NA	16 (100)	16 (100)
		R	NA		0
10	Chloramphenicol (CAF)	S	11(68.75)	16 (100)	16 (100)
		I	3(18.75)	NA	NA
		R	2(12.5)	0	0
11	Linezolid (LZD)	S	NA	16 (100)	16 (100)
		R	NA	16 (100)	16 (100)
12	Cefotaxime (FOT)	S	NA	0	0
		R	NA		
13	Tetracycline (TET)	S	1(6.25)	1(6.25)	1(6.25)
		I	1(6.25)	NA	NA
		R	14(87.5)	15(93.75)	15(93.75)
14	Cefoxitin (CXT)	S	NA	16 (100)	16 (100)
		R	NA	0	0
15	Azitromycin (AZM)	S	13 (81.25)		
		I	1(6.25)	NA	NA
		R	2(12.5)		

MIC-Minimum inhibitory concentration by using Broth Dilution Technique, WGS - Whole Genome Sequencing, NA - Not applicable

## Discussion

In this study, serotype II represented by ST-10 and ST-569, belongs to CC-10 was the most prevalent colonizer of newborns. It is in congruent with that of a study conducted in Taiwan where serotype II was the most colonizer,4/11 (33.3%) [36]. However, it is different from a study conducted before three decades in the same hospital where 60% were type Ib followed by type Ia (16%) [37]; in Poland, serotype III (35%) was the most prevalent types followed by Ia (20%), V (17%), II (15%), Ib (8%) and IV (5%) [38]. Similarly, a study from Iran showed that serotype III (50%) was the most common serotype, followed by serotype II (25%), Ia (12%), V (11%), and Ib (2%) [39].

Regarding the MLST analysis, we found that the most predominant ST was ST-10 which is quite different from various reports across the world. For instance, a study in Iran showed ST-19 (34.6%), ST-28(21.0%), ST-335, ST-12 and ST-8 each with 10.5% [40], in Japan, ST-17 (29.3%), ST-23 (17.3%0, and ST-19916.0%) [41], and in Sweden ST-19 (23.0%), ST-17 (17.2%0, and ST-23 (17.2%) [42]. The variation observed might be attributed to the geographical variations, the type of study participants involved in which certain studies isolated invasive GBS while in our case is the colonizing GBS isolated from recto-vaginal swabs of pregnant women and body surfaces of newborns. This variation also might be attributed to the limited number of GBS isolates sequenced in our study. The most predominant CCs we found was CC-10 followed by CC-19, which is slightly congruent with the reports from Poland where CC-23 (49.0%), CC-19 (17.0%), and CC-10 and CC-17 each with 10.0% [43], and in Taiwan, CC-1 (28.0%), CC-12 (26.0%) and CC-19 (18.0%), CC-17 (14.0%), and CC-23 (12.0%) [44].

In our study, ALPH, SRR and Pilli were identified. Of the ALPH families, ALPHA was found to be the predominant surface protein followed by ALP1. All our isolates carried SRR1, a glycoprotein that may play an important role in crossing the blood brain-barrier (BBB) and for the development of streptococcal meningitis [45, 46]. Serine-rich repeat proteins also help to promote initial contact of GBS with host epithelium, cervical and vaginal attachment [47] which result in GBS cervicovaginal colonization and provide a survival benefit in the vaginal mucosa. In addition, three forms of pilus proteins (PI-1 / PI-2A1, PI-1 / PI-2B, and PI-1 / PI-2A3) were identified in our study. A study showed that pilus vaccine containing the PI-1, PI-2a, and PI-2b elicited good opsonophagocytic antibodies that confer protection in mice and this suggested that pilus components of GBS are highly immunogenic [48, 49]. It implies that increasing our knowledge about the profiles of the pilus island (PI) among GBS isolates obtained from different geographic locations is useful for future efforts that aimed at the development of maternal GBS vaccines from bacterial protein components (pilus-based), which were effective in animal models. This variable presence of specific PIs has considerable implications for the development of maternal GBS vaccines which target these bacterial proteins.

The drug susceptibility patterns of the 16 GBS isolates tested by BDI and WGS seem to resemble what is seen in other countries, such as studies conducted in Iran [40] and Poland [43] showed that, all GBS isolates were susceptible to penicillin, vancomycin, and linezolid, Improving the laboratory facilities for routine antibiotic susceptibility tests, mainly in developing countries, should be mandatory.

The prevalent tetracycline resistance was caused by the presence of the TETL.TETM genes. In agreement with our results, a study described that tetracycline resistance was predominantly due to tetM (83.7%) or along with tetL (12.2%) [50]. Another study conducted in Italy also revealed that the tetracycline resistance rate was 62/91 (68.1%), all resistant isolates harbored the *tet*(M) gene [21]. On the same way, resistant to tetracycline was observed in all 19 (100%) strains and 18/19 (98.0%) was correlated with presence of the tetM gene in a study carried out in Iran [40]. Tetracycline resistance is due to acquisition of *tet* determinant that encodes for antibiotic efflux or ribosomal protection in gram positive cocci [51, 52].

### Limitation

The limitation of this study was that the numbers of the GBS isolates sequenced were small.

## Conclusion

In this study, the MLST data reveals that the study isolates are of different phylogeny and is suggestive of different clones circulating in Ethiopia. WGS data showed that tetM and tetL / tetM were found as the tetracycline resistant determinant genes in 93.8% of the GBS isolates. All other antibiotics tested for by using MIC and WGS methods were susceptible. We found that penicillin is still the drug of choice for maternal GBS IAP. Vaccine formulation would include serotype II, V, Ia, and VI, besides serotype Ib, and III to give broader coverage of GBS prevention across different geographical locations including the study area particularly and in the globe generally since serotype VI was also often reported from the Asian studies. Further studies on serotype and molecular epidemiology will provide a comprehensive data of the GBS capsular serotypes, sequence types, clones, surface proteins and drug resistance determinant genes available in Ethiopia.

## Supplementary information

**Additional file 1:** Whole genome sequencing (WGS) and Minimum inhibitory concentration (MIC) by using broth dilution method test results of colonizing GBS.

## Data Availability

All data generated or analyzed during this study are included in this published article [and its supplementary information file, S1].

## Abbreviations

ALP: Alpha-Like Protein
BDT: Broth Dilution Test
CC: Clonal Complex
CDC: Centers for Disease Control and Prevention
CLSI: Clinical Laboratory Standard Institute
CPS: Capsular Polysaccharide
EOD: Early Onset Disease
Erm: Erythromycin resistance methylase
GBS: Group B Streptococcus
IAP: Intrapartum Antibiotic Prophylaxis
LOD: Late Onset Disease
Mef: Macrolide efflux protein
MIC: Minimum Inhibitory Concentration
MLS_B_: Macrolides, Lincosamides, and Streptogramin_B_
MLST: Multi-Locus Sequence Type
PI: *Pilus Island*
Srr: Serine-rich repeat
ST: Sequence Type
TETL.TETM/: Tetracycline resistance determinant protein
WGS: Whole Genome Sequencing
