# Supplementary material for: Molecular characterization of Streptococcus agalactiae isolated from pregnant women and newborns at the University of Gondar Comprehensive Specialized Hospital, Northwest Ethiopia
Source: BMC Infect Dis. 2020 Jan 13;20:35. doi: 10.1186/s12879-020-4776-7 (PMC6958622; doi:10.1186/s12879-020-4776-7)
Supplement: Supplementary file 1 — Additional file 1: Whole genome sequencing (WGS) and Minimum inhibitory concentration (MIC) by using broth dilution method test results of colonizing GBS. [file 12879_2020_4776_MOESM1_ESM.docx]

**Supplement information file (S1)**

**Whole genome sequencing (WGS) and Minimum inhibitory concentration (MIC) test results (broth dilution method) of colonizing GBS**

Table1. Minimum inhibitory concentration (MIC) test results of colonizing GBS by using broth dilution test method (n=12), 2019

| LABID | ProjectID | Species | Pathogen | AMP | FOX | CLI | CIP | CZL | TAX | TETSIGN | TET | DAPSIGN | DAP | ERYSIGN | ERY | LFX |
| --- | --- | --- | --- | --- | --- | --- | --- | --- | --- | --- | --- | --- | --- | --- | --- | --- |
| 1451-19 | Ethiopia | 3 | GBS | 0.25 | 8 | 0.12 | 2 | 0.5 | 0.12 | > | 8 | <= | 0.5 | <= | 0.06 | 2 |
| 1469-19 | Ethiopia | 3 | GBS | 0.25 | 8 | 0.12 | 2 | 0.5 | 0.12 | > | 8 | <= | 0.5 | <= | 0.06 | 2 |
| 1471-19 | Ethiopia | 3 | GBS | 0.25 | 8 | 0.12 | 2 | 0.5 | 0.12 | > | 8 | = | 1 | <= | 0.06 | 1 |
| 1477-19 | Ethiopia | 3 | GBS | 0.25 | 8 | 0.12 | 1 | 0.5 | 0.12 | > | 8 | = | 1 | <= | 0.06 | 1 |
| 1479-19 | Ethiopia | 3 | GBS | 0.25 | 8 | 0.12 | 2 | 0.5 | 0.12 | > | 8 | = | 1 | <= | 0.06 | 2 |
| 1522-19 | Ethiopia | 3 | GBS | 0.25 | 8 | 0.12 | 2 | 0.5 | 0.12 | > | 8 | <= | 0.5 | <= | 0.06 | 2 |
| 1536-19 | Ethiopia | 3 | GBS | 0.25 | 8 | 0.12 | 2 | 0.5 | 0.12 | = | 0.5 | = | 1 | <= | 0.06 | 2 |
| 1546-19 | Ethiopia | 3 | GBS | 0.25 | 8 | 0.12 | 2 | 0.5 | 0.12 | > | 8 | <= | 0.5 | <= | 0.06 | 1 |
| 1568-19 | Ethiopia | 3 | GBS | 0.25 | 8 | 0.12 | 1 | 0.5 | 0.12 | > | 8 | <= | 0.5 | <= | 0.06 | 1 |
| 1570-19 | Ethiopia | 3 | GBS | 0.25 | 8 | 0.12 | 2 | 0.5 | 0.12 | > | 8 | = | 1 | <= | 0.06 | 1 |
| 1582-19 | Ethiopia | 3 | GBS | 0.25 | 8 | 0.12 | 2 | 0.5 | 0.12 | > | 8 | = | 1 | <= | 0.06 | 2 |
| 1621-19 | Ethiopia | 3 | GBS | 0.25 | 8 | 0.12 | 2 | 0.5 | 0.12 | > | 8 | = | 1 | <= | 0.06 | 2 |
|  |  |  |  |  |  |  |  |  |  |  |  |  |  |  |  |  |
| Table 1: Continued | | | | | | | | | | | | | | | | |
| LABID | ProjectID | Species | Pathogen | LZOSIGN | LZO | PEN | LZOSIGN | LZO | PEN | LZOSIGN | LZO | PEN | LZOSIGN | LZO | PEN | LZOSIGN |
| 1451-19 | Ethiopia | 3 | GBS | <= | 1 | 0.12 | <= | 1 | 0.12 | <= | 1 | 0.12 | <= | 1 | 0.12 | <= |
| 1469-19 | Ethiopia | 3 | GBS | <= | 1 | 0.12 | <= | 1 | 0.12 | <= | 1 | 0.12 | <= | 1 | 0.12 | <= |
| 1471-19 | Ethiopia | 3 | GBS | <= | 1 | 0.12 | <= | 1 | 0.12 | <= | 1 | 0.12 | <= | 1 | 0.12 | <= |
| 1477-19 | Ethiopia | 3 | GBS | <= | 1 | 0.12 | <= | 1 | 0.12 | <= | 1 | 0.12 | <= | 1 | 0.12 | <= |
| 1479-19 | Ethiopia | 3 | GBS | <= | 1 | 0.12 | <= | 1 | 0.12 | <= | 1 | 0.12 | <= | 1 | 0.12 | <= |
| 1522-19 | Ethiopia | 3 | GBS | <= | 1 | 0.12 | <= | 1 | 0.12 | <= | 1 | 0.12 | <= | 1 | 0.12 | <= |
| 1536-19 | Ethiopia | 3 | GBS | <= | 1 | 0.12 | <= | 1 | 0.12 | <= | 1 | 0.12 | <= | 1 | 0.12 | <= |
| 1546-19 | Ethiopia | 3 | GBS | <= | 1 | 0.12 | <= | 1 | 0.12 | <= | 1 | 0.12 | <= | 1 | 0.12 | <= |
| 1568-19 | Ethiopia | 3 | GBS | <= | 1 | 0.12 | <= | 1 | 0.12 | <= | 1 | 0.12 | <= | 1 | 0.12 | <= |
| 1570-19 | Ethiopia | 3 | GBS | <= | 1 | 0.12 | <= | 1 | 0.12 | <= | 1 | 0.12 | <= | 1 | 0.12 | <= |
| 1582-19 | Ethiopia | 3 | GBS | <= | 1 | 0.12 | <= | 1 | 0.12 | <= | 1 | 0.12 | <= | 1 | 0.12 | <= |
| 1621-19 | Ethiopia | 3 | GBS | <= | 1 | 0.12 | <= | 1 | 0.12 | <= | 1 | 0.12 | <= | 1 | 0.12 | <= |
|  |  |  |  |  |  |  |  |  |  |  |  |  |  |  |  |  |

Table2. Serotype, clonality and allelic profiles of GBS housekeeping genes obtained by using whole genome sequencing (WGS) (n=16), 2019

| **Original SampleID** | **LABID** | **WGS_serotype** | **CC** | **ST** | **adhP** | **pheS** | **atr** | **glnA** | **sdhA** | **glcK** | **tkt** |  |  |  |  |
| --- | --- | --- | --- | --- | --- | --- | --- | --- | --- | --- | --- | --- | --- | --- | --- |
| RV4 | 1451-19 | V | 19 | 2 | 1 | 1 | 3 | 1 | 1 | 2 | 2 |  |  |  |  |
| RV68 | 1451-20 | V | 19 | 2 | 1 | 1 | 3 | 1 | 1 | 2 | 2 |  |  |  |  |
| RV383 | 1451-21 | V | 19 | 2 | 1 | 1 | 3 | 1 | 1 | 2 | 2 |  |  |  |  |
| RV61 | 1451-22 | II | 10 | 10 | 9 | 1 | 4 | 1 | 3 | 3 | 2 |  |  |  |  |
| RV222 | 1451-23 | II | 10 | 10 | 9 | 1 | 4 | 1 | 3 | 3 | 2 |  |  |  |  |
| N246 | 1451-24 | II | 10 | 10 | 9 | 1 | 4 | 1 | 3 | 3 | 2 |  |  |  |  |
| N254 | 1451-25 | II | 10 | 10 | 9 | 1 | 4 | 1 | 3 | 3 | 2 |  |  |  |  |
| N267 | 1451-26 | II | 10 | 10 | 9 | 1 | 4 | 1 | 3 | 3 | 2 |  |  |  |  |
| U223 | 1451-27 | II | 10 | 10 | 9 | 1 | 4 | 1 | 3 | 3 | 2 |  |  |  |  |
| E100 | 1451-28 | VI | 1 | 14 | 1 | 1 | 2 | 1 | 5 | 2 | 2 |  |  |  |  |
| RV73 | 1451-29 | II | 10 | 569 | 9 | 1 | 1 | 1 | 3 | 1 | 2 |  |  |  |  |
| RV83 | 1451-30 | II | 10 | 569 | 9 | 1 | 1 | 1 | 3 | 1 | 2 |  |  |  |  |
| RV89 | 1451-31 | II | 10 | 569 | 9 | 1 | 1 | 1 | 3 | 1 | 2 |  |  |  |  |
| RV94 | 1451-32 | II | 10 | 569 | 9 | 1 | 1 | 1 | 3 | 1 | 2 |  |  |  |  |
| E83 | 1451-33 | II | 10 | 569 | 9 | 1 | 1 | 1 | 3 | 1 | 2 |  |  |  |  |
| RV269 | 1451-34 | IA | 23 | 933 | 5 | 4 | 6 | 1 | 2 | 1 | 82 |  |  |  |  |
|  |  |  |  |  |  |  |  |  |  |  |  |  |  |  |  |

Table3: Whole genome sequencing based antibiotic susceptibility results of colonizing GBS (n=16), 2019

| **Original SampleID** | **LABID** | **PBP1A** | **PBP2X** | **WGS_ZOX_SIGN** | **WGS_ZOX_MIC** | **WGS_ZOX_SIR** | **WGS_FOX_SIGN** | **WGS_FOX_MIC** | **WGS_FOX_SIR** | **WGS_TAX_SIGN** | **WGS_TAX_MIC** | **WGS_TAX_SIR** | **WGS_CFT_SIGN** | **WGS_CFT_MIC** | **WGS_CFT_SIR** | **WGS_CPT_SIGN** | **WGS_CPT_MIC** | **WGS_CPT_SIR** |
| --- | --- | --- | --- | --- | --- | --- | --- | --- | --- | --- | --- | --- | --- | --- | --- | --- | --- | --- |
| RV4 | 1451-19 | NF | NF |  |  |  |  |  |  |  |  |  |  |  |  |  |  |  |
| RV68 | 1451-20 | 1 | 1 | <= | 0.5 | U | <= | 8 | U | <= | 0.12 | S | <= | 0.12 | S | <= | 0.12 | S |
| RV383 | 1451-21 | 1 | 1 | <= | 0.5 | U | <= | 8 | U | <= | 0.12 | S | <= | 0.12 | S | <= | 0.12 | S |
| RV61 | 1451-22 | 1 | 1 | <= | 0.5 | U | <= | 8 | U | <= | 0.12 | S | <= | 0.12 | S | <= | 0.12 | S |
| RV222 | 1451-23 | 1 | 1 | <= | 0.5 | U | <= | 8 | U | <= | 0.12 | S | <= | 0.12 | S | <= | 0.12 | S |
| N246 | 1451-24 | 1 | 1 | <= | 0.5 | U | <= | 8 | U | <= | 0.12 | S | <= | 0.12 | S | <= | 0.12 | S |
| N254 | 1451-25 | 1 | 1 | <= | 0.5 | U | <= | 8 | U | <= | 0.12 | S | <= | 0.12 | S | <= | 0.12 | S |
| N267 | 1451-26 | 1 | 1 | <= | 0.5 | U | <= | 8 | U | <= | 0.12 | S | <= | 0.12 | S | <= | 0.12 | S |
| U223 | 1451-27 | 1 | 1 | <= | 0.5 | U | <= | 8 | U | <= | 0.12 | S | <= | 0.12 | S | <= | 0.12 | S |
| E100 | 1451-28 | 1 | 1 | <= | 0.5 | U | <= | 8 | U | <= | 0.12 | S | <= | 0.12 | S | <= | 0.12 | S |
| RV73 | 1451-29 | 1 | 1 | <= | 0.5 | U | <= | 8 | U | <= | 0.12 | S | <= | 0.12 | S | <= | 0.12 | S |
| RV83 | 1451-30 | 1 | 1 | <= | 0.5 | U | <= | 8 | U | <= | 0.12 | S | <= | 0.12 | S | <= | 0.12 | S |
| RV89 | 1451-31 | 1 | 1 | <= | 0.5 | U | <= | 8 | U | <= | 0.12 | S | <= | 0.12 | S | <= | 0.12 | S |
| RV94 | 1451-32 | 1 | 1 | <= | 0.5 | U | <= | 8 | U | <= | 0.12 | S | <= | 0.12 | S | <= | 0.12 | S |
| E83 | 1451-33 | 1 | 1 | <= | 0.5 | U | <= | 8 | U | <= | 0.12 | S | <= | 0.12 | S | <= | 0.12 | S |
| RV269 | 1451-34 | 1 | 5 | <= | 0.5 | U | <= | 8 | U | <= | 0.12 | S | <= | 0.12 | S | <= | 0.12 | S |
|  |  |  |  |  |  |  |  |  |  |  |  |  |  |  |  |  |  |  |

Table3: Antibiotics susceptibility test results Continued

| **Original SampleID** | **LABID** | **WGS_CZL_SIGN** | **WGS_CZL_MIC** | **WGS_CZL_SIR** | **WGS_AMP_SIGN** | **WGS_AMP_MIC** | **WGS_AMP_SIR** | **WGS_PEN_SIGN** | **WGS_PEN_MIC** | **WGS_PEN_SIR** | **WGS_MER_SIGN** | **WGS_MER_MIC** | **WGS_MER_SIR** | **WGS_TET** | **WGS_TET_SIGN** | **WGS_TET_MIC** | **WGS_TET_SIR** | **WGS_EC** |
| --- | --- | --- | --- | --- | --- | --- | --- | --- | --- | --- | --- | --- | --- | --- | --- | --- | --- | --- |
| RV4 | 1451-19 |  |  |  |  |  |  |  |  |  |  |  |  | TETM | >= | 8 | R | neg |
| RV68 | 1451-20 | <= | 0.5 | U | <= | 0.25 | S | <= | 0.12 | S | <= | 0.12 | S | TETM | >= | 8 | R | neg |
| RV383 | 1451-21 | <= | 0.5 | U | <= | 0.25 | S | <= | 0.12 | S | <= | 0.12 | S | TETM | >= | 8 | R | neg |
| RV61 | 1451-22 | <= | 0.5 | U | <= | 0.25 | S | <= | 0.12 | S | <= | 0.12 | S | TETL:TETM | >= | 8 | R | neg |
| RV222 | 1451-23 | <= | 0.5 | U | <= | 0.25 | S | <= | 0.12 | S | <= | 0.12 | S | TETL:TETM | >= | 8 | R | neg |
| N246 | 1451-24 | <= | 0.5 | U | <= | 0.25 | S | <= | 0.12 | S | <= | 0.12 | S | TETL:TETM | >= | 8 | R | neg |
| N254 | 1451-25 | <= | 0.5 | U | <= | 0.25 | S | <= | 0.12 | S | <= | 0.12 | S | TETL:TETM | >= | 8 | R | neg |
| N267 | 1451-26 | <= | 0.5 | U | <= | 0.25 | S | <= | 0.12 | S | <= | 0.12 | S | TETL:TETM | >= | 8 | R | neg |
| U223 | 1451-27 | <= | 0.5 | U | <= | 0.25 | S | <= | 0.12 | S | <= | 0.12 | S | TETL:TETM | >= | 8 | R | neg |
| E100 | 1451-28 | <= | 0.5 | U | <= | 0.25 | S | <= | 0.12 | S | <= | 0.12 | S | TETM | >= | 8 | R | neg |
| RV73 | 1451-29 | <= | 0.5 | U | <= | 0.25 | S | <= | 0.12 | S | <= | 0.12 | S | TETM | >= | 8 | R | neg |
| RV83 | 1451-30 | <= | 0.5 | U | <= | 0.25 | S | <= | 0.12 | S | <= | 0.12 | S | TETM | >= | 8 | R | neg |
| RV89 | 1451-31 | <= | 0.5 | U | <= | 0.25 | S | <= | 0.12 | S | <= | 0.12 | S | TETM | >= | 8 | R | neg |
| RV94 | 1451-32 | <= | 0.5 | U | <= | 0.25 | S | <= | 0.12 | S | <= | 0.12 | S | TETM | >= | 8 | R | neg |
| E83 | 1451-33 | <= | 0.5 | U | <= | 0.25 | S | <= | 0.12 | S | <= | 0.12 | S | TETM | >= | 8 | R | neg |
| RV269 | 1451-34 | <= | 0.5 | U | <= | 0.25 | S | <= | 0.12 | S | <= | 0.12 | S | neg | <= | 2 | S | neg |
|  |  |  |  |  |  |  |  |  |  |  |  |  |  |  |  |  |  |  |

Table3: Antibiotics susceptibility test results Continued

| **Original SampleID** | **LABID** | **WGS_ERY_SIGN** | **WGS_ERY_MIC** | **WGS_ERY_SIR** | **WGS_CLI_SIGN** | **WGS_CLI_MIC** | **WGS_CLI_SIR** | **WGS_LZO_SIGN** | **WGS_LZO_MIC** | **WGS_LZO_SIR** | **WGS_SYN_SIGN** | **WGS_SYN_MIC** | **WGS_SYN_SIR** | **WGS_ERYCLI** | **WGS_FQ** | **WGS_CIP_SIGN** | **WGS_CIP_MIC** | **WGS_CIP_SIR** | **WGS_LFX_SIGN** |
| --- | --- | --- | --- | --- | --- | --- | --- | --- | --- | --- | --- | --- | --- | --- | --- | --- | --- | --- | --- |
| RV4 | 1451-19 | <= | 0.25 | S | <= | 0.25 | S | <= | 2 | S | <= | 1 | S | neg | neg | <= | 2 | U | <= |
| RV68 | 1451-20 | <= | 0.25 | S | <= | 0.25 | S | <= | 2 | S | <= | 1 | S | neg | neg | <= | 2 | U | <= |
| RV383 | 1451-21 | <= | 0.25 | S | <= | 0.25 | S | <= | 2 | S | <= | 1 | S | neg | neg | <= | 2 | U | <= |
| RV61 | 1451-22 | <= | 0.25 | S | <= | 0.25 | S | <= | 2 | S | <= | 1 | S | neg | neg | <= | 2 | U | <= |
| RV222 | 1451-23 | <= | 0.25 | S | <= | 0.25 | S | <= | 2 | S | <= | 1 | S | neg | neg | <= | 2 | U | <= |
| N246 | 1451-24 | <= | 0.25 | S | <= | 0.25 | S | <= | 2 | S | <= | 1 | S | neg | neg | <= | 2 | U | <= |
| N254 | 1451-25 | <= | 0.25 | S | <= | 0.25 | S | <= | 2 | S | <= | 1 | S | neg | neg | <= | 2 | U | <= |
| N267 | 1451-26 | <= | 0.25 | S | <= | 0.25 | S | <= | 2 | S | <= | 1 | S | neg | neg | <= | 2 | U | <= |
| U223 | 1451-27 | <= | 0.25 | S | <= | 0.25 | S | <= | 2 | S | <= | 1 | S | neg | neg | <= | 2 | U | <= |
| E100 | 1451-28 | <= | 0.25 | S | <= | 0.25 | S | <= | 2 | S | <= | 1 | S | neg | neg | <= | 2 | U | <= |
| RV73 | 1451-29 | <= | 0.25 | S | <= | 0.25 | S | <= | 2 | S | <= | 1 | S | neg | neg | <= | 2 | U | <= |
| RV83 | 1451-30 | <= | 0.25 | S | <= | 0.25 | S | <= | 2 | S | <= | 1 | S | neg | neg | <= | 2 | U | <= |
| RV89 | 1451-31 | <= | 0.25 | S | <= | 0.25 | S | <= | 2 | S | <= | 1 | S | neg | neg | <= | 2 | U | <= |
| RV94 | 1451-32 | <= | 0.25 | S | <= | 0.25 | S | <= | 2 | S | <= | 1 | S | neg | neg | <= | 2 | U | <= |
| E83 | 1451-33 | <= | 0.25 | S | <= | 0.25 | S | <= | 2 | S | <= | 1 | S | neg | neg | <= | 2 | U | <= |
| RV269 | 1451-34 | <= | 0.25 | S | <= | 0.25 | S | <= | 2 | S | <= | 1 | S | neg | neg | <= | 2 | U | <= |
|  |  |  |  |  |  |  |  |  |  |  |  |  |  |  |  |  |  |  |  |

Table3: Antibiotics susceptibility test results Continued

| **Original SampleID** | **LABID** | **WGS_LFX_MIC** | **WGS_LFX_SIR** | **WGS_Other** | **WGS_DAP_SIGN** | **WGS_DAP_MIC** | **WGS_DAP_SIR** | **WGS_VAN_SIGN** | **WGS_VAN_MIC** | **WGS_VAN_SIR** | **WGS_RIF_SIGN** | **WGS_RIF_MIC** | **WGS_RIF_SIR** | **WGS_CHL_SIGN** | **WGS_CHL_MIC** | **WGS_CHL_SIR** | **WGS_COT_SIGN** | **WGS_COT_MIC** | **WGS_COT_SIR** |
| --- | --- | --- | --- | --- | --- | --- | --- | --- | --- | --- | --- | --- | --- | --- | --- | --- | --- | --- | --- |
| RV4 | 1451-19 | 2 | S | neg | <= | 1 | S | <= | 1 | S | <= | 1 | U | <= | 4 | S | <= | 0.5 | U |
| RV68 | 1451-20 | 2 | S | neg | <= | 1 | S | <= | 1 | S | <= | 1 | U | <= | 4 | S | <= | 0.5 | U |
| RV383 | 1451-21 | 2 | S | neg | <= | 1 | S | <= | 1 | S | <= | 1 | U | <= | 4 | S | <= | 0.5 | U |
| RV61 | 1451-22 | 2 | S | neg | <= | 1 | S | <= | 1 | S | <= | 1 | U | <= | 4 | S | <= | 0.5 | U |
| RV222 | 1451-23 | 2 | S | neg | <= | 1 | S | <= | 1 | S | <= | 1 | U | <= | 4 | S | <= | 0.5 | U |
| N246 | 1451-24 | 2 | S | neg | <= | 1 | S | <= | 1 | S | <= | 1 | U | <= | 4 | S | <= | 0.5 | U |
| N254 | 1451-25 | 2 | S | neg | <= | 1 | S | <= | 1 | S | <= | 1 | U | <= | 4 | S | <= | 0.5 | U |
| N267 | 1451-26 | 2 | S | neg | <= | 1 | S | <= | 1 | S | <= | 1 | U | <= | 4 | S | <= | 0.5 | U |
| U223 | 1451-27 | 2 | S | neg | <= | 1 | S | <= | 1 | S | <= | 1 | U | <= | 4 | S | <= | 0.5 | U |
| E100 | 1451-28 | 2 | S | neg | <= | 1 | S | <= | 1 | S | <= | 1 | U | <= | 4 | S | <= | 0.5 | U |
| RV73 | 1451-29 | 2 | S | neg | <= | 1 | S | <= | 1 | S | <= | 1 | U | <= | 4 | S | <= | 0.5 | U |
| RV83 | 1451-30 | 2 | S | neg | <= | 1 | S | <= | 1 | S | <= | 1 | U | <= | 4 | S | <= | 0.5 | U |
| RV89 | 1451-31 | 2 | S | neg | <= | 1 | S | <= | 1 | S | <= | 1 | U | <= | 4 | S | <= | 0.5 | U |
| RV94 | 1451-32 | 2 | S | neg | <= | 1 | S | <= | 1 | S | <= | 1 | U | <= | 4 | S | <= | 0.5 | U |
| E83 | 1451-33 | 2 | S | neg | <= | 1 | S | <= | 1 | S | <= | 1 | U | <= | 4 | S | <= | 0.5 | U |
| RV269 | 1451-34 | 2 | S | neg | <= | 1 | S | <= | 1 | S | <= | 1 | U | <= | 4 | S | <= | 0.5 | U |
|  |  |  |  |  |  |  |  |  |  |  |  |  |  |  |  |  |  |  |  |

Table4: surface protein profiles of colonizing GBS obtained by using Whole genome sequencing(n=16), 2019

| **Original SampleID** | **LABID** | **ALPH** | **SRR** | **Pili** | **HVGA** | **Flow_cell** | **Instrument** | **Retest** | **Retest_result** | **Comment** | **Isolation_year** | **Contig_num** | **N50** | **Longest_contig** | **Total_bases** |
| --- | --- | --- | --- | --- | --- | --- | --- | --- | --- | --- | --- | --- | --- | --- | --- |
| RV4 | 1451-19 | ALP1 | SRR1 | PI1:PI2A1 | neg | C5B4P | M06079 | yes | done | panel | Ethiopia_Mucheye |  |  |  |  |
| RV68 | 1451-20 | ALP1 | SRR1 | PI1:PI2A1 | neg | C5B4P | M06079 |  |  |  | Ethiopia_Mucheye | 36 | 154151 | 389473 | 2123519 |
| RV383 | 1451-21 | ALP1 | SRR1 | PI1:PI2A1 | neg | C5B4P | M06079 |  |  |  | Ethiopia_Mucheye | 29 | 136443 | 389493 | 2126782 |
| RV61 | 1451-22 | ALPHA | SRR1 | PI1:PI2A1 | neg | C5B4P | M06079 |  |  |  | Ethiopia_Mucheye | 60 | 85692 | 179712 | 2049638 |
| RV222 | 1451-23 | neg | SRR1 | PI1:PI2A1 | neg | C5B4P | M06079 |  |  |  | Ethiopia_Mucheye | 71 | 74284 | 133693 | 2031351 |
| N246 | 1451-24 | neg | SRR1 | PI1:PI2A1 | neg | C5B4P | M06079 |  |  |  | Ethiopia_Mucheye | 100 | 65164 | 179058 | 2065816 |
| N254 | 1451-25 | neg | SRR1 | PI1:PI2A1 | neg | C5B4P | M06079 |  |  |  | Ethiopia_Mucheye | 78 | 82248 | 179131 | 2033318 |
| N267 | 1451-26 | neg | SRR1 | PI1:PI2A1 | neg | C5B4P | M06079 |  |  |  | Ethiopia_Mucheye | 1797 | 6059 | 100613 | 3891488 |
| U223 | 1451-27 | ALPHA | SRR1 | PI1:PI2A1 | neg | C5B4P | M06079 |  |  |  | Ethiopia_Mucheye | 58 | 85697 | 179087 | 2043421 |
| E100 | 1451-28 | ALP1 | SRR1 | PI1:PI2B | neg | C5B4P | M06079 |  |  |  | Ethiopia_Mucheye | 29 | 137184 | 345589 | 2116176 |
| RV73 | 1451-29 | ALPHA | SRR1 | PI1:PI2A1 | neg | C5B4P | M06079 |  |  |  | Ethiopia_Mucheye | 40 | 150703 | 331697 | 2022186 |
| RV83 | 1451-30 | ALPHA | SRR1 | PI1:PI2A1 | neg | C5B4P | M06079 |  |  |  | Ethiopia_Mucheye | 36 | 111901 | 180879 | 2018612 |
| RV89 | 1451-31 | ALPHA | SRR1 | PI1:PI2A1 | neg | C5B4P | M06079 |  |  |  | Ethiopia_Mucheye | 279 | 108514 | 180729 | 2086254 |
| RV94 | 1451-32 | ALPHA | SRR1 | PI1:PI2A1 | neg | C5B4P | M06079 |  |  |  | Ethiopia_Mucheye | 40 | 138563 | 224711 | 2018343 |
| E83 | 1451-33 | ALPHA | SRR1 | PI1:PI2A1 | neg | C5B4P | M06079 |  |  |  | Ethiopia_Mucheye | 36 | 150759 | 332123 | 2023055 |
| RV269 | 1451-34 | ALP23 | SRR1 | PI1:PI2A3 | neg | C5B4P | M06079 |  |  |  | Ethiopia_Mucheye | 97 | 166116 | 486267 | 2082078 |
|  |  |  |  |  |  |  |  |  |  |  |  |  |  |  |  |
